# Trait Hostility and Acute Inflammatory Responses to Stress in the Laboratory

**DOI:** 10.1371/journal.pone.0156329

**Published:** 2016-06-06

**Authors:** Dominique Girard, Jean-Claude Tardif, Julie Boisclair Demarble, Bianca D’Antono

**Affiliations:** 1 Research Center, Montreal Heart Institute, Montreal, Quebec, Canada; 2 Department of Psychology, Université du Québec à Montréal, Montreal, Quebec, Canada; 3 Department of Medicine, Université de Montréal, Montreal, Quebec, Canada; 4 Department of Psychology, Université de Montréal, Montreal, Quebec, Canada; University of Kansas Medical Center, UNITED STATES

## Abstract

Hostility has been associated with higher basal levels of inflammation. The present study evaluated the association of hostility with acute stress-induced changes in inflammatory activity. One hundred and ninety-nine healthy men and women, aged 19–64 years, were exposed to a stress protocol involving four interpersonal stressors. Participants completed the Cook-Medley Hostility questionnaire and provided two blood samples for the measurement of inflammatory biomarkers (CRP, Il-6, MPO, TNF-α, MCP-1, Il-8, Il-10, and Il-18), prior to and following exposure to a standardized stress protocol. In univariate analyses, hostility was associated with significantly higher TNF-α, but lower Il-8 and Il-18 values post-stress, though only Il-8 remained significant after controlling for baseline differences. In multivariate analyses, a significant Age by Hostility interaction emerged for Il-6, while sex moderated the relation between hostility and Il-10 reactivity. Following stress, hostility was associated with greater pro-inflammatory Il-6 activity among younger individuals and to decreased anti-inflammatory Il-10 activity in women. Future research is needed to replicate these findings and to evaluate their implication for disease.

## Introduction

Hostility, a multidimensional concept involving anger, quarrelsomeness, or tendencies toward cynicism, mistrust and denigration of others [1], has been shown to increase risk for coronary artery disease (CAD) morbidity and mortality [2,3]. The mechanisms underlying this increased risk for CAD are still unclear, though altered inflammatory activity in more hostile individuals may be involved [4–7].

Inflammation has been of increased interest given its role in the development and progression of atherosclerosis in CAD [8,9]. Hansson [8] describes, for example, the development of an inflammatory cascade in atherosclerosis, involving, but not limited to, inflammatory cytokines (e.g. tumor necrosis factor alpha (TNF-α), interleukin-1 (Il-1)), interleukin-6 (Il-6), and acute-phase reactants (e.g. C-reactive protein (CRP)).

A small but growing body of mostly cross-sectional studies has shown elevations in CRP, Il-6, and/or TNF-α in more hostile individuals [4–7,10–13]. However, inconsistent results have emerged [10,11,14] suggesting that sample characteristics, such as age and sex may influence the relation observed between hostility and inflammatory activity. Moreover, the mechanisms involved in bringing about these elevations in inflammatory activity in more hostile individuals have been the subject of little research to date. It has been demonstrated that more hostile individuals show heightened neurohumoral or cardiovascular reactivity to stress [15,16] and this heightened reactivity to stress may contribute to higher blood levels of inflammatory markers [17].

However, little is known about whether more hostile individuals actually show greater inflammatory responses to acute stress compared to less hostile individuals. Kiecolt-Glaser et al. [18] examined this in 42 healthy married couples. Hostility was measured with the Rapid Marital Interaction Coding System, and blood samples (for TNF-α and Il-6 assays) were obtained prior to, as well as 24-hours following exposure to interpersonal stress. Hostile couples did not show significantly greater increases in Il-6 or TNF-α 24 hours post baseline compared to low hostile couples. In contrast, in another study involving 525 healthy male and female siblings, more hostile individuals did show an increase in CRP (but not Il-6) levels one hour following exposure to an acute laboratory stress protocol involving two 5-minute emotion recall tasks [19]. Brydon et al. [13], for their part, examined the relation between hostility and Il-6 reactivity to stress in 34 men who survived an acute coronary syndrome. Participants were exposed to a stress protocol involving a 5-min *stroop* color-word interference task and a public speaking task. Blood samples were obtained at baseline as well as 30, 75 and 120 minutes after the completion of the final task. Hostility was associated with increased Il-6 levels 75 and 120 minutes after the stress protocol compared to baseline. Finally, a recent study exposed 140 individuals with type 2 diabetes to a stress protocol involving the stroop color-word interference task and mirror tracing [20]. Plasma Il-6 was measured at baseline, during the 2 stress tasks and 45 and 75 minutes post stress. Hostile individuals showed greater increases in Il-6 following, but not during the stress tasks.

Thus, only four studies have examined inflammatory responses to acute stress in the laboratory, with mixed results. These investigations were limited to one or two inflammatory markers (CRP, Il-6, or TNF-α). However, other inflammatory markers also play a significant role in the development of CAD. For example, monocyte chemotactic protein 1 (MCP-1) and myeloperoxidase (MPO) are both involved in the formation and progression of atherosclerotic plaque [21,22]. The former is a protein responsible for monocyte and T-cell migration into the vessel wall [22,23]. MPO is a hemoprotein released from neutrophils and monocytes during inflammation, and is implicated in lipid oxidation promoting arthogenesis [24,25]. Pro-inflammatory cytokines Il-8 and Il-18 also play a role in triggering atherosclerosis [26,27]. Il-10, on the other hand, has an anti-inflammatory role [28] and may protect against age-related increases in levels of Il-6, oxidative stress, and endothelial dysfunction [29]. Examining differential patterns of results across various inflammatory markers may provide indices as to the underlying mechanisms linking hostility to increased risk of CAD.

Finally, personal characteristics, such as sex and age, may moderate the impact of hostility on stress-induced changes in inflammatory activity, but this was examined in only one study. It was found that depressive symptoms, but not hostility, led to an increase in CRP and Il-6 one hour following an emotion-recall protocol among women but not men [19]. Moreover, an overview of the literature suggests that age may influence the association between hostility and inflammation, with less consistent relations observed in studies performed in older individuals [5,14,30]. Importantly, we have previously reported that positive relations between hostility and basal levels of TNF-α, Il-6 and CRP were particularly evident among younger individuals and among women in the current sample [10].

In the present analyses, we examined whether hostility is associated with greater acute inflammatory responses to psychological stress in the laboratory and whether these relations were moderated by sex and age. Eight inflammatory markers were assessed, including TNF-α, Il-6, CRP, MPO, MCP-1, as well as interleukins 8, 10, and 18, to examine the specificity of associations with hostility. Based on previous results from this laboratory regarding basal inflammatory levels [10], we expected that more hostile individuals would show greater changes in inflammatory activity in response to acute stress, and that this would be particularly evident among women and younger individuals.

## Materials and Methods

This study was part of a larger investigation we conducted on the relation between psychophysiological factors and intermediary risk for cardiovascular disease [10,31–38].

### Participants

Healthy working men (n = 81) and women (n = 118) aged 19–64 years (*M* = 41.4, *SD* = 11.5) were recruited via advertisements in newspapers and community centers in the greater Montreal area. Eligibility criteria included (a) no utilization of mental health services within the past year, (b) no current or known health problems (hypertension, diabetes, hypercholesterolemia, heart disease, cancer, autoimmune disorders, adrenal disorders, etc.) or use of medication (statins, beta-blockers, anti-inflammatory agents, etc.) with possible effects on cardiovascular, immune, or neuroendocrine functions, (c) no learning or cognitive disabilities that could impair the capacity to complete questionnaires or follow instructions and (d) not currently on hormone replacement therapy. The recruitment was done so as to obtain an equal representation across the entire age range of 18–65 years. Women were over-sampled to ensure sufficient numbers of menopausal women needed for a separate objective of the study not examined here. Participants with CRP values greater than 10 mg/L, suggestive of potential acute infection, were excluded post-hoc. Due to loss of samples as a result of technical difficulties (n = 25) and presence of severe outliers, data were available for 187 participants for CRP, Il-6, MPO, and TNF-α and 160 participants for MCP-1, Il-10, Il-8, and Il-18.

### Procedure

Eligible participants came to a scheduled appointment at the Montreal Heart Institute at 8:00 AM on a weekday in order to control for circadian rhythms in physiological activity. Participants were asked to abstain from drinking (other than water), eating, doing exercise and smoking for 12 hours prior to the appointment and from drinking alcohol or consuming drugs for 24 hours. If these conditions were not met on the day of the testing, or if participants presented physical symptoms such as a cough, a new appointment was scheduled.

In the laboratory, participants interacted with a same-sex research assistant, trained to maintain a neutral tone and expression throughout testing. Anthropomorphic data were obtained and electrodes placed for electrocardiogram monitoring on the participant’s lower rib cage, in a bipolar configuration with a ground electrode on the left hip. A cuff was placed on the non-dominant arm for blood pressure monitoring. Participants were asked to complete socio-demographic, medical and psychological questionnaires. A 10-minute rest-period followed during which baseline physiological measures were recorded. A first blood draw and the stress tasks ensued.

The stress protocol involved four interpersonal challenges; a public reading task, two role-playing tasks, and a non-scripted debate. Each task lasted 5 minutes and were preceded by a 5-minute tape-recorded autogenic relaxation period and a 2-minute preparation phase and followed by a 5-minute recovery period. One of the objectives of the overall study was to examine sex/age differences in reactivity to the role-plays as a function of hostility. Therefore, relaxation procedures were introduced to minimize carry-over effects of one stressor onto the next. A second blood draw was taken at the end of the final recovery period. Twenty-four hour ambulatory blood pressure and ECG data were obtained following the laboratory session. Participants received a $200 Canadian compensation for time and travel. The Research and Ethics Board of the Montreal Heart Institute approved this study. Free and informed written consent was obtained prior to study onset.

#### Laboratory tasks

All four tasks have led to significant affective and physiological reactivity in prior studies [36,39,40], and their efficacy in inducing stress across emotional, cardiovascular and autonomic measures were demonstrated in the current study as well (for details, see [32]). Participants were videotaped throughout the protocol and told that their performance would be rated to increase motivation and stressfulness of the tasks.

#### Public reading task

This task consisted of reading a neutral text about Antarctica’s geography in front of the research assistant.

#### Role-plays

Following the neutral reading task, the participants performed two validated scripted role-plays that manipulated hostile behavior. Participants were required to enact a situation in which they were a personnel supervisor giving feedback to an employee who has not performed well at work. The research assistant played the role of the employee to whom the feedback was provided. In one condition, the feedback given was based on agreeable comments, such as “I can see you tried hard. We just have to work on the parts of the task that you did not perform so well on.” In the second condition, the participants use quarrelsome behaviors when giving their feedback (e.g. “I think that a high-school freshman could do better than this. I am not impressed by your performance.”). The participants were told to enact the role as faithfully as possible. The order of presentation of the role-plays was counterbalanced across participants.

#### Debate

The final task was a non-scripted debate on the legality of abortion. The participants chose their position on the issue and debated it with the research assistant for 1-minute periods at a time. The participant always started first, resulting in 3 minutes of speaking and 2 minutes of listening. A fact sheet was provided to the participant once his position was chosen in order to help him/her prepare the debate.

### Measurements

#### Socio-demographic variables

Sex, age, marital status, annual income and years of schooling were included.

#### Health information

Health information relating to tobacco, caffeine, and alcohol consumption as well as physical activity was obtained.

#### Hostility

The Cook-Medley Hostility Inventory (CMHo; [41]) is an extensively used self-report questionnaire composed of 50 true-false items that measures tendencies toward cynicism, hostile affect, and aggressive responding. The internal consistency of this instrument (α = 0.82–0.86) [41,42] and test-retest reliability (rs > 0.85) [43] are excellent. In the current sample, the internal consistency was α = 0.83.

#### The Beck Depression Inventory-II (BDI-II; [44]) and the Social Support Questionnaire [45]

BDI-II and the Social Support Questionnaire measured with an adaptation of the MOS Social Support Survey [45] respectively measured depression symptoms and social support and were included here as potential covariates where pertinent. In several investigations, depressive symptoms were shown to confound the relation between hostility and inflammatory activity [19,30,46,47]. Low social support has similarly been associated with increased inflammatory levels [48–50].

#### Blood samples

Blood samples were analyzed using validated assays. CRP was measured from serum using the Siemens (formerly Dade Behring) CardioPhase hsCRP assay (Siemens Healthcare Diagnostics Products GmbH, Marburd, Germany) on the BN ProSpec Nephelometer (Siemens Healthcare Diagnostics Products GmbH). The minimal detectable hsCRP concentration was 0.18 mg/L.

Il-6 was measured from serum using the R&D Systems Quantikine High Sensitivity Il-6 ELISA assay (Car. NO. HS600B, R&D Systems, Minneapolis, USA). We used the smaller standard (0.156ng/L) as the sensitivity level.

TNF-α was measured from serum, using the R&D System Quantikine High Sensitivity TNF-α ELISA assay (Cat. No. HSTA00D, R&D Systems, Minneapolis, USA). The smaller standard (0,5 ng/L) was used as the sensitivity level.

MPO was measured from plasma using the ALPCO Diagnostics Myeloperoxidase (MPO) ELIZA assay (Revised version, Cat. No. 30-6631A, ALPCO Diagnostics, Salem, NH, USA). The sensitivity level was fixed with the smaller standard (1.9μg/L).

Il-8, Il-10, Il-18 and MCP-1 measurements were obtained from plasma using the Bio-Plex Protein Array System with two Bio-Plex Human Cytokine Panels (Bio-Rad, Hercules, CA). A sample dilution of 1:4 was used to analyze these biomarkers. A Bio-Plex human 4-Plex cytokine assay kit (Bio-Rad Laboratories, Hercules, CA, USA) was used to assay for samples for the presence of Il-8 and MCP-1. A second Bio-Plex human 2-Plex cytokine assay kit (Bio-Rad Laboratories-Hercules, CA, USA) was used for measuring Il-10 and Il-18. The smaller standard was used as sensitivity level: 0.6 ng/L for Il-8, 0.68 ng/L for MCP-1, 0.93 ng/L for Il-10, and 0.78 ng/L for Il-18.

### Additional biomarkers considered as covariates or for posthoc analyses

#### Testosterone

Testosterone was extracted from plasma by ethyl ether. Testosterone was measured by ELISA according to the manufacturer’s instructions (Noegen Corporation, MI, USA). Male samples were diluted 100 times in extraction buffer before being tested, whereas female samples were diluted 10 times. Each sample was analyzed in duplicate.

#### Estradiol and FSH

Estradiol was measured from serum by electrochemiluminescence immunoassay using the Roche TSH assay (Roche Diagnostics, Mannheim, Germany) on the Elecsys 2010 analyzer (Roche Diagnostics). FSH was measured from serum by electrochemiluminescence immunoassay using the Roche TSH assay (Roche Diagnostics, Mannheim, Germany) on the Elecsys 2010 analyzer (Roche Diagnostics). This assay utilizes two monoclonal antibodies in a sandwich format.

#### Salivary cortisol

Cortisol was measured from saliva using salivettes (Sarstedt, Montreal, Canada) containing a piece of absorbent gauze. Participants had to chew on the swab for 45 seconds until it was saturated with saliva. The correlate-EIA enzyme immunoassay kit was used to analyze samples off-site (for more details, please see [51]).

#### Systolic and diastolic blood pressure (BP)

BP was measured using Accutor Plus automated blood pressure monitor (Datascope Inc., Montvale, MJ) with a standard inflatable cuff placed on the participant’s nondominant arm. This model uses an oscillometric method and has been recommended by the European Society of Hypertension [52]. A mean of two readings per period was used for analysis. For baseline BP, the last 5 minutes of the 10 minute baseline period prior to the first blood draw was used.

#### Heart rate variability (HRV)

Spectral analysis of HRV was performed off-line using Fast Fourier Transformations of the interbeat intervals (RR) in MATLAB using published algorithms [53,54] that characterize the high frequency (HF; 0.15-.040 Hz) and the low frequency (LF; 0.04–0.15 Hz) as recommended by the Task Force of the European Society of Cardiology and the North American Society of Pacing and Electrophysiology (1996). For more information about HRV measurements, please see Dragomir et al. [32].

#### Metabolic burden

Metabolic burden consists of the number of metabolic parameters for which participants were in the higher quartile (lower for HDL) for their sex. Measures for HDL-cholesterol, triglycerides, glucose, waist circumference, and BP were considered (NCEP-ATP III, 2004). Lipids and glucose in heparinized plasma samples were assayed using respective reagent Flex on the multianalyzer Dimension RxL Max (Dade Behring Diagnostics, Marburg, Germany) as soon as possible following blood draw. 24-hour ambulatory monitoring was used for BP. The measures were taken every 20 minutes in the daytime and every hour from 22h00 to 6h00, using Spacelab Ambulatory Blood Pressure Units. Ambulatory blood pressure monitoring has been found to be more reliable than clinic or laboratory measures [51]. An average value over 24 hours was taken as a measure of BP.

### Preliminary analyses

Natural logarithmic transformations were applied to CRP, Il-6, TNF-α, Il-10, Il-8 and Il-18 to normalize their distribution.

#### Univariate correlations

Pearson correlations were used to examine relations between hostility and each inflammatory value obtained post-stress (uncorrected and corrected for the baseline value). Correlations between each post-stress inflammatory value and potential covariates were also examined. Potential covariates were chosen based on their association with inflammatory activity in the literature. We considered socio-demographic, psychological (social support, BDI-II), medical (BMI, metabolic burden) and health behavior variables. Variables were included as covariates when they correlated at *p* ≤ 0.10 with changes in inflammatory values.

#### Evaluation of sex and age differences in the relation between hostility and stress-induced changes in inflammatory activity

The potential moderating role of sex and age were examined using hierarchical linear regressions. The dependent variable was the post-stress inflammatory value. Block 1 included age, sex, the baseline inflammatory value, and relevant covariates. Hostility was forced into block 2 and its two- and three-way interactions with age and sex were entered stepwise in Block 3. Examination of the post-stress values controlling for the baseline values was chosen instead of change scores to facilitate interpretation of the results. This approach has the added advantage of further controlling for the impact of baseline differences on stress-induced changes in inflammation.

Statistical significance was set at *p <* .05. When the interaction effects were significant, simple slope analyses were performed using lower and higher estimates for age and hostility based on values ±1 SD from the mean [55]. When interaction terms were significant, lower order interactions or main effects were not interpreted as per recommendations. No significant collinearity was observed in the analyses.

## Results

### Sample characteristics

Table 1 presents participants’ socio-demographic and behavioral characteristics separately for women and men. As women were over-sampled for menopausal status they were slightly older, on average, than men. Men also exercised more on average per week while women presented slightly higher scores on the BDI-II.

**Table 1:** Socio-demographic and behavioural profile of participants.

Characteristics	Men (n = 81)	Women (n = 118)
	Men (n = 81)	Women (n = 118)
Demographic variables		
Age (years)*	39.37 (11.3)	42.83 (11.38)
Body mass index (kg/m^2^)	24.83 (4.06)	25.3 (5.60)
Years of schooling	15.83 (3.43)	15.95 (3.47)
Married/living with someone	35(43)	46(39)
Annual family income n (%)		
≤$29 999	27(33)	40(34)
$30 000–59 999	25(31)	47(40)
≥$60 000	29(36)	31(26)
Behavioral/ medical variables		
Smoker n (%)	13(16)	29(25)
Cigarettes/Week	9.49 (28.65)	13.58 (32.32)
# Caffeine beverages	2.69 (6.66)	1.60 (4.68)
Hours of exercise/week**	4.61 (5.21)	2.51 (3.182)
Metabolic burden	1.37 (1.35)	1.45 (1.32)
Psychological variables		
Social support	20.68 (5.09)	20.63 (5.28)
BDI-II*	7.17 (7.06)	9.25 (7.37)
CMHo	20.02 (7.64)	18.09 (7.66)

BDI-II, Beck Depression Inventory II; CMHo, Cook-Medley Hostility Inventory.

*P < .05

**P < .01

Table 2 presents the levels of the inflammatory markers prior to and after the stress protocol. In the overall sample, participants exhibited a significant decrease in circulating levels of MCP-1 and Il-8 in response to the stress tasks, but no other significant change was observed.

**Table 2:** Mean (and SD) inflammatory blood levels pre- and post-stress.

		Pre-stress		Post-stress	
Markers	N	M	SD	M	SD
CRP (mg/L)	187	1.423	1.828	1.420	1.849
IL-6 (ng/L)	187	1.035	1.062	0.989	1.093
TNF-alpha (ng/L)	187	1.277	0.704	1.253	0.725
MPO (μg/L)	187	62.559	18.319	63.271	18.317
MCP-1 (ng/L)***	160	42.061	18.588	35.933	16.306
Il-8 (ng/L)**	160	3.462	2.589	3.270	2.660
Il-10 (ng/L)	160	1.338	1.415	1.266	0.972
Il-18 (ng/L)	160	77.408	38.621	75.120	35.955

CRP, C Reactive Protein; IL, Interleukin; MPO, myeloperoxidase; TNF-α, Tumor Necrosis Factor–alpha; MCP-1, Monocyte Chemotactic Protein 1.

**P < .01

***P < .001

### Univariate correlations between cynical hostility and inflammatory activity

Cynical hostility was associated with significantly greater TNF-α but lower MCP-1 and Il-8 activity post-stress. Hostility also showed a positive, though non-significant, trend with Il-6, CRP, and Il-18 (*p* < .10). However, when correcting for original baseline differences, only the negative association with Il-8 remained significant (Table 3).

**Table 3:** Univariate correlations (and *P* values) between cynical hostility and inflammatory post-stress levels.

	Cynical Hostility	
Biomarker	Uncorrected for Baseline value	Corrected for Baseline value
CRP	0.123(0.093)	0.048(0.514)
Il-6	0.140(0.057)	0.017(0.816)
MPO	0.054(0.497)	-0.023(0.760)
TNF-α	0.148(0.043)*	-0.033(0.654)
MCP-1	-0.170(0.032)*	-0.047(0.555)
Il-8	-0.160(0.043)*	-.159(0.046)*
Il-10	-0.029(0.716)	-0.088(0.269)
Il-18	0.144(0.069)	0.044(0.579)

CRP, C Reactive Protein; Il, Interleukins; MPO, myeloperoxidase; TNF-α, Tumor Necrosis Factor–alpha; MCP-1, Monocyte Chemotactic Protein 1.

*P < .05

### Multivariate associations of cynical hostility with post-stress inflammatory activity and moderating effects of sex and age

#### Il-6 (Table 4)

**Fig 1:**
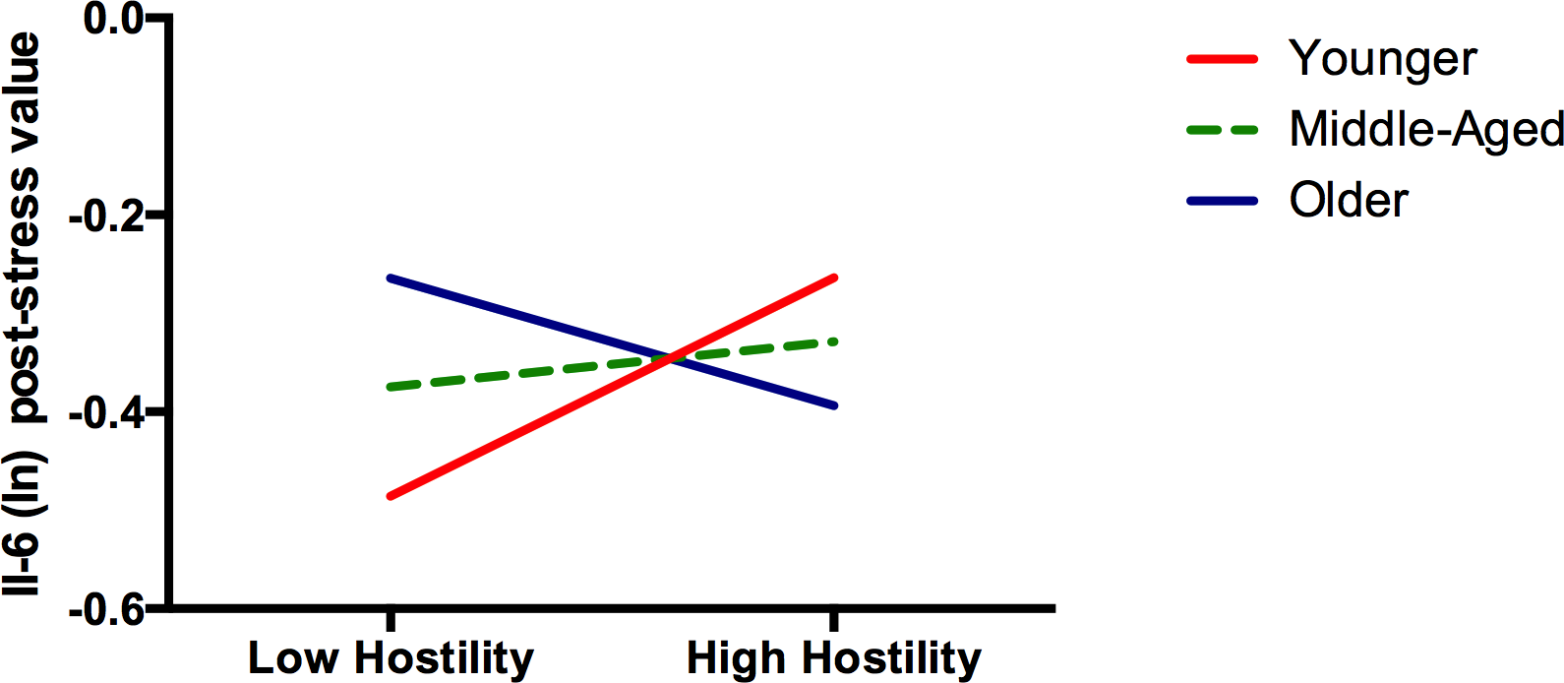
The Relation between Hostility and Il-6 Is Moderated by Age. Simple slope analyses indicate that among younger individuals, hostility was associated with greater Il-6 concentrations post stress compared to low hostile individuals (younger, *b* = 0.014, *P* = 0.001), whereas among older individuals, the opposite was true (*b* = -0.008, *P* = 0.047). In the intermediate age group, no significant relation was observed (b = 0.003, *P* = 0.338).

A significant Age by Hostility interaction emerged in Model 3, as well as a significant Sex by Age by Hostility interaction in Model 4. However, when all lower level interactions were forced in, the 3-way interaction was no longer significant. For the sake of parsimony, only the significant Age by Hostility interaction was retained. Results from simple slope analyses indicated that post-stress, cynical hostility was related to higher Il-6 concentrations among younger individuals (b = .014, *p* = .001), but to lower levels among older individuals (b = -.008, *p* = .047) (Fig 1).

**Table 4:** Summary of multivariate associations between hostility and post-stress Il-6 levels.

Final Model	β	t	*P*
Age	0.029	0.874	0.106
Sex	0.004	0.128	0.898
BMI	0.048	1.251	0.213
# People cohabiting	0.018	0.593	0.554
Educational status	0.045	1.480	0.141
# Drinks of alcohol/week	-0.046	-1.473	0.143
# Caffeinated drinks/week	-0.025	-0.790	0.431
Metabolic Burden	0.070	1.723	0.087
Baseline Il-6 level	0.858	24.446	<0.001
CMHo	0.031	0.993	0.322
Age*CMHo	-0.065	-2.168	0.031
F_model_ (11, 175) = 91.134, *P*<0. 001			
R^2^_model_ = 0.851, R^2^_adj_ = 0.842			

BMI, Body Mass Index; CMHo, Cook Medley Hostility Inventory.

#### Il-10 (Table 5)

**Fig 2:**
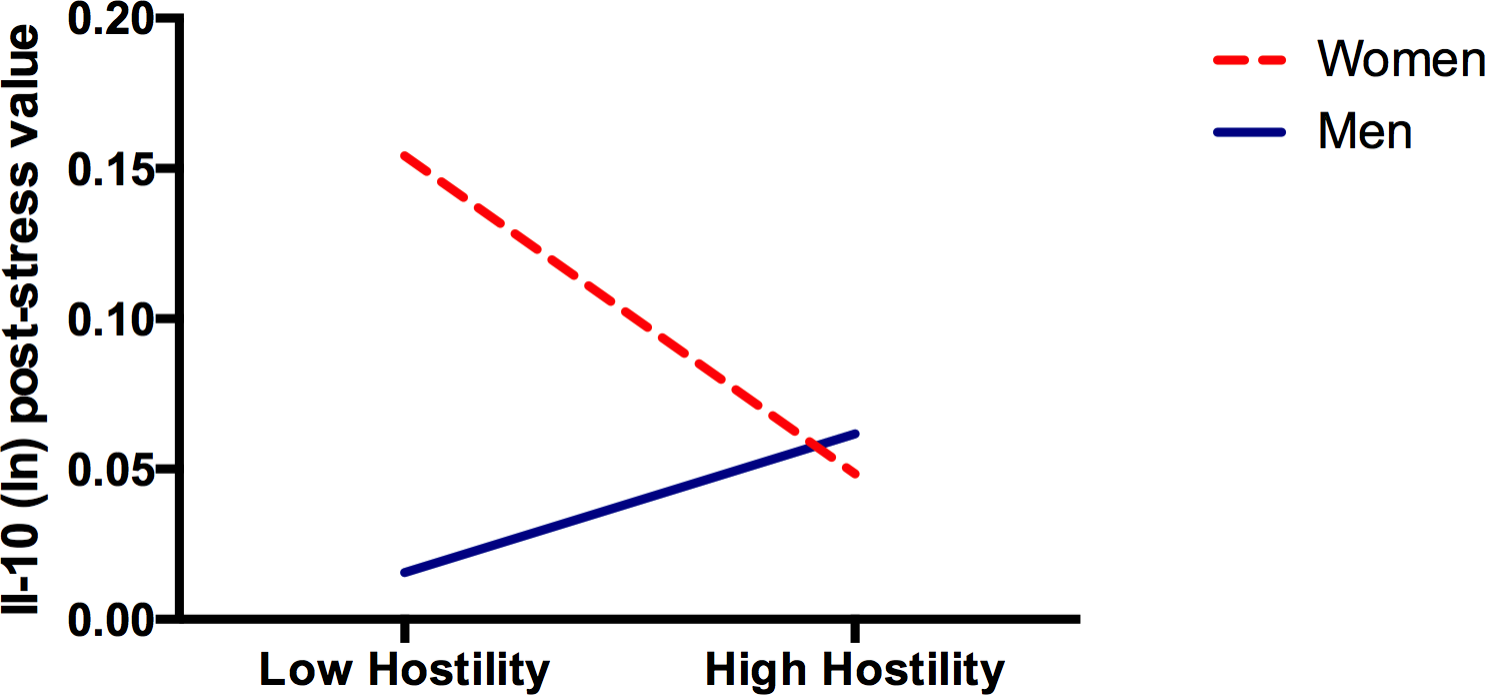
The Relation between Hostility and Il-10 is Moderated by Sex. In women, greater hostility is associated with significantly reduced Il-10 post-stress (*b* = -0.019, *P* = 0.038). In men, hostility was not significantly associated with post-stress Il-10 values (*b* = 0.005, *P* = 0.437).

A significant Sex by Hostility interaction emerged. Hostility was associated with significantly reduced post-stress Il-10 concentrations in women (b = -.013, *p* = .038), but not men (b = .005, *p* = .437) (Fig 2).

**Table 5:** Summary of multivariate associations between hostility and post-stress Il-10 levels.

Final Model	β	t	*P*
Age	0.041	0.653	0.541
Sex	0.026	0.429	0.669
Personal Income	-0.109	-1.782	0.077
Baseline Il-10 value	0.658	11.055	<0.001
CMHo	-0.056	-0.935	0.351
Sex*CMHo	-0.120	-2.013	0.046
F_model_ (7, 153) = 19.833, *P<*0.001			
R^2^_model_ = 0.476, R^2^_adj_ = 0.452			

CMHo, Cook Medley Hostility Inventory. The Sex*CMHo interaction explained an additional 1.4% of variance in post-stress Il-10.

No significant main or interaction effects emerged for the remaining variables (Tables 6–11).

**Table 6:** Multivariate analysis of associations between hostility and post-stress CRP levels.

CRP	β	t	*P*
CMHo	0.007	0.997	0.320
F_model_ (9, 177) = 3085.425, *P*<0.001			
R^2^_model_ = 0.994, R^2^_adj_ = 0.993			

*Notes*. Analyses controlled for age, sex, BMI, #alcoholic drinks per week, number of hours of exercise per week, Beck Depression Inventory II scores, metabolic burden and baseline CRP values. These accounted for 99.4% of the variance. Baseline CRP values accounted for 56% of the variance on its own.

**Table 7:** Multivariate analysis of associations between hostility and post-stress MPO levels.

MPO	β	t	*P*
CMHo	-0.011	-0.410	0.682
F_model_ (4, 182) = 306.828, *P*<0.001			
R^2^_model_ = 0.871, R^2^_adj_ = 0.868			

*Notes*. Analyses controlled for age, sex, BMI, baseline MPO values, and accounted for 87% of the variance.

**Table 8:** Multivariate analysis of associations between hostility and post-stress MCP-1 levels.

MCP-1	β	t	*P*
CMHo	-0.010	-0.276	0.783
F_model_ (6, 153) = 107.533, *P*<0.001			
R^2^_model_ = 0.808 R^2^_adj_ = 0.801			

*Notes*. Analyses controlled for age, sex, baseline MCP-1 values, marital status, and number of children. Covariates accounted for 81% of the variance (baseline MCP-1 values accounted for 76% of the variance on its own).

**Table 9:** Multivariate analysis of associations between hostility and post-stress TNF-α levels.

TNF-α	β	t	*P*
CMHo	-0.003	-0.091	0.928
F_model_ (10, 176) = 106.653, *P*<0.001			
R^2^_model_ = 0.858, R^2^_adj_ = 0.850			

*Notes*. Analyses controlled for age, sex, BMI, metabolic burden, # of alcoholic drinks per week, number of children, annual family income, Beck Anxiety Inventory scores, and baseline TNF-α values. These accounted for 86% of the variance (69% accounted for by baseline values).

**Table 10:** Multivariate analysis of associations between hostility and post-stress Il-8 levels.

Il-8	β	t	*P*
CMHo	-0.068	-1.879	0.062
F_model_ (5, 154) = 128.993, *P*<0.001			
R^2^_model_ = 0.807 R^2^_adj_ = 0.801			

*Notes*. Analyses controlled for age, sex, number of children, and baseline Il-8 values; and accounted for 80% of the variance. Baseline Il-8 values accounted for 76% of the variance in post-stress values.

**Table 11:** Multivariate analysis of associations between hostility and post-stress Il-18 levels.

Il-18	β	t	*P*
CMHo	0.009	0.262	0.793
F_model_ (7, 152) = 121.748, *P*<0.001			
R^2^_model_ = 0.849 R^2^_adj_ = 0.843			

*Notes*. Analyses controlled for age, sex, #cigarette smoked per week, years of schooling, metabolic burden, and baseline Il-18 values; and accounted for 85% of the variance. Baseline Il-18 values accounted for 78% of the variance in post-stress values.

### Post-hoc analyses

Given the purported regulatory role of Il-10 on cytokine activity, analyses for post-stress Il-6 values were repeated in the 160 participants for whom complete data were available and controlled additionally for Il-10 change scores to examine to what extent individual differences in Il-10 response to stress might mediate the age differences in Il-6 variation. Analyses on this reduced sample revealed a non-significant trend for the 2-way Age by Hostility interaction which was only slightly reduced after controlling for the change in Il-10 (ß = -.063, *p* = .055 vs. ß = -.061, *p* = .063).

Sex and age differences in sex hormone levels have been hypothesized to contribute to differences in hostility and in inflammatory activity. Additional analyses were performed to examine their potential role in our results. Testosterone was inversely correlated with age (r = -.228, *p <* .01), sex (r = -.744, *p <* .001) and with the Il-6 change score (r = -.136; *p <* .05). Controlling for testosterone levels only slightly reduced significance for the Il-10 Sex by Hostility interaction (ß = -0.116, *p* = .053) but led to no change in the Age by Hostility interaction for Il-6.

As female sex hormones were assayed only in women, sub-analyses including these variables were performed only in women. Respectively, follicular stimulating hormone (FSH) and estrogen were correlated with age (r = .669, *p <* .001; r = -.547, *p <* .001) and the Il-6 change score (r = -.214, *p <* .05; r = .167, *p <* .05), but not with Il-10 change score. When controlling for FSH and estradiol hormones, hostility continued to show a negative trend with post-stress Il-10 values (ß = -.158, *p* = .065) in women. The Age by Hostility interaction for Il-6 similarly approached significance (ß = -.064, *p* = .092). Lack of significance was likely due to reduced power in this smaller subsample. Thus individual differences in sex hormones appear to play only a limited role in the effects of age or sex on the cytokine results.

It has been suggested that autonomic and neuroendocrine responses to stress could influence inflammatory reactions [56]. Stress-induced changes in systolic blood pressure (SBP) (r = -.200) and diastolic blood pressure (DBP) (r = -.220) were significantly correlated with Il-10 change score. Similarly, stress-induced changes in DBP (r = -.202) and mean arterial blood pressure (MAP) (r = -.213), were significantly and negatively correlated with Il-6 change score. SBP change scores showed a similar trend (r = -.136, *p* = .063). HRV and cortisol measures did not show strong associations with either the Il-6 or Il-10 change scores (all *p*s > 0.100). When controlling for stress-induced changes in BP, the Sex by Hostility interaction for Il-10 remained significant (ß = -.136, *p* = .021), as did the Age by Hostility interaction for Il-6 (ß = -.060, *p* = .048).

## Discussion

This study sought to examine whether cynical hostility is associated with altered inflammatory responses to a standardized stress protocol, and whether these relations are influenced by age and/or sex, in a broad array of inflammatory biomarkers. In univariate analyses, hostility was associated with significantly greater circulating post-stress levels of TNF-α, with similar trends for CRP, Il-6, and Il-18. However, these results reflected the association of these markers with basal variations, as control for baseline values rendered these associations non-significant. Surprisingly, hostility was associated with lower levels of Il-8 and MCP-1 post-stress. For Il-8, the negative association remained significant after controlling for baseline variations. This is the first investigation to our knowledge to observe negative associations between some markers of inflammation and hostility. Our findings also extend existing literature by showing that sex and age moderate the relation between hostility and acute Il-6 and Il-10 responses to stress. More specifically, cynical hostility was associated with greater post-stress Il-6 concentrations among younger individuals whereas the opposite was observed among older individuals. Moreover, hostility was associated with significantly lower levels of the anti-inflammatory biomarker Il-10 post-stress among women but not men.

Brummett et al. [19] and Kiecolt-Glaser et al. [18] reported no significant association between hostility and stress-induced increases in levels of Il-6 compared to baseline in healthy samples of men and women. In contrast, and consistent with our own findings of greater Il-6 concentrations post-stress among younger more hostile individuals, Brydon et al. [13] showed hostility to be associated with increased Il-6 levels following a laboratory stress protocol in a male-only sample of acute coronary syndrome survivors. Hackett et al. [20] reported similar findings in patients with diabetes. Of note, however, is the fact that in these two latter studies, Il-6 increased following the stress protocol irrespective of hostility. This was not the case for all individuals in the current investigation.

Moreover, we did not observe hostility to influence stress responses across the measures of CRP, MPO, TNF-α, MCP-1, Il-8 and Il-18 in multivariate analyses that controlled for baseline elevations. Findings with TNF-α are consistent with those of Kiecolt-Glaser et al. [18]. Conversely, Brummett et al. [19], in their study of siblings, had observed an increase in CRP as a function of hostility. No research was found on the impact of hostility on stress-induced changes in the other inflammatory markers measured in our study.

Differences in results obtained across studies may reflect methodological differences in the sample characteristics, types of stressors used, the measure of hostility, and the timing of the blood samples. For example, Brydon et al. [13] and Hackett et al. [20] showed main effects of hostility on stress-induced inflammatory changes in patients with CAD or diabetes, while this was not consistently observed in healthier samples. Hackett et al. [20] suggest that hostility may be particularly detrimental in individuals at greater risk for CAD morbidity or mortality. Our results did not reflect lack of stressfulness of our protocol as participants showed considerable cardiovascular, neuroendocrine, and autonomic responses to the tasks [32–34,40]. Moreover, other studies have similarly found no significant change in inflammation levels following a stress protocol [57,58]. For example, Heesen et al. [57] reported no change in Il-6 immediately after a 45-minute stress protocol involving a mental arithmetic task, the Stroop color-word interference test, and a public-speaking task, in both multiple sclerosis and healthy participants. Neurohumoural changes may have minimized inflammatory responses via the stimulation of glucocorticoids [59] and of the autonomic nervous system [59,60]. Glucocorticoids suppress pro-inflammatory cytokines (e.g. Il-6, TNF-α), but up-regulate anti-inflammatory cytokines (e.g. Il-4 and Il-10) [59]. Cathecholamines, neurotransmitters playing a role in the sympathetic nervous system, also show anti-inflammatory functions [60–63]. It was found, for example, that the activation of β_2_-adrenergic receptors by norepinephrine dampened expression of TNF-α [60] and IFNgamma, a pro-inflammatory cytokine and significantly enhanced production of Il-10 [63]. Similarly, in the current investigation, stress exposure led to decreased Il-8 and MCP-1 concentrations, but controlling for stress-induced neurohumoral changes did not alter the results.

The timing of the post-stress venipuncture may have led to underestimation of the impact of stress (and of hostility) on inflammatory activity. Indeed, blood samples were obtained approximately 10 minutes following the end of the stress protocol (approximately 70 minutes after the onset of the first stressor). These various biomarkers have different activity peaks, although the specific peak time following stimulation for each marker is still largely unknown and appears dependent on the source of stimulation. Il-6 has shown stronger effects following acute stress, when blood testing is delayed 30–120 minutes post-stress [64]. For example, Prather et al. [65] found that immediately after stress, Il-6 concentrations decreased significantly in men, but not women. Thirty minutes later, however, increases in Il-6 blood levels were observed in both healthy middle-aged men and women. Peters et al. [66] found similar results in healthy young men. Thus, at this stage, results from the current study can only be generalized to acute inflammatory responses to interpersonal stress. Additional research examining delayed responses to stress is necessary to better characterize the influence of hostility on stress-induced changes in inflammatory activity, as well as the pertinence of these changes to disease processes.

While some researchers have observed that acute stress can promote anti-inflammatory activity (e.g. Il-10, Il-19) [67,68] and suppress pro-inflammatory activity (e.g. Il-1β, Il-6, TNF-α) [66,67], others have instead reported decreased anti-inflammatory Il-4 and Il-10 concentrations following a stress task among men and women [69,70]. The latter findings are concordant with our own Il-10 results among more hostile women in the current study. It has been suggested that increases in Il-10 might protect against age-related increases in Il-6, oxidative stress and endothelial dysfunction [29], and confer protection for the immune system against the inflammatory stress response [71]. As such, the absence of this compensatory mechanism in more hostile women may render them more vulnerable to the pathogenic effects of stress. Nonetheless, post-hoc analyses of Il-6 controlling for change in Il-10 suggested but a mitigated role for this anti-inflammatory marker in the Il-6 results, at least as measured concurrently. More research is required on sex and hostility differences in Il-10 and its ability to compensate for stress-induced inflammatory responses.

Various factors may have contributed to the current results. Sex hormones, for example, may influence hostility as well as inflammatory activity. Indeed, higher testosterone levels have been shown to correlate with greater hostility [72] and, as was the case in this study, with decreased pro-inflammatory activity [73,74]. Female sex steroids have also been shown to correlate negatively with pro-inflammatory activity [75]. However, post-hoc analyses controlling for sex hormones suggest that these played only a limited role in the age and sex differences observed in Il-6 and Il-10 activity in the current study.

Separate research has shown, greater stress-induced changes in neuroendocrine and autonomic activity in more hostile individuals [16,76] and it has been hypothesized that enhanced reactivity to stress in these physiological systems may be responsible for their greater inflammatory activity [17]. While stress-induced changes in blood pressure were indeed associated with subsequent decreases in both Il-6 and Il-10, these changes were not responsible for the hostility related findings obtained in this study. At this time, it is unclear what factors may be driving these sex and age differences in the relation between hostility and acute stress-induced changes in inflammatory activity.

Current findings must be considered in the context of certain limitations. First, our participants were mainly French-speaking Caucasians. Generalizability to individuals of other ethnic backgrounds is thus uncertain. Moreover, the participants (young and older alike) were healthy and may represent a rather resilient group. More specifically, individuals more vulnerable to the effects of hostility on their health may have already developed disease states (or died from them), effectively excluding them from participation in our study. This may have reduced our ability to detect significant associations between hostility and changes in acute inflammation response to stress. In addition, several analyses were performed, which may have increased the possibility of false positive results. Given the exploratory nature of this study, it was deemed premature to enforce control for multiple testing. However, our findings with Il-6 and Il-10 changes are consistent with some existing literature, and with prospective data from this sample showing elevations in Il-6 following a three-year period among more hostile individuals (manuscript in preparation). As mentioned previously, post-stress blood samples were obtained but once, approximately 10 minutes following the end of the stress protocol. Additional samples over a longer follow-up period may have revealed a different pattern of results. Finally, depressive symptoms were shown to moderate the relation between hostility and inflammatory activity in several studies. For example, Brummett et al. [19] reported that individuals who reported elevations in both depressive symptoms and hostility showed the largest CRP response to a psychological stress protocol. A similar result was obtained by Stewart et al. [30]. The moderating effect of depression on the relation between hostility and inflammatory responses to stress was not examined here as it was not the objective of this investigation. Important, however, was the fact that results were maintained when depressive symptoms were covaried. Nonetheless, given these limitations, results can only be considered hypothesis generating at this time.

On the other hand, the sample was heterogeneous with respect to age, sex, education, income, and type of work, increasing the extent to which data may be generalizable to at least (French-speaking) Caucasians. Several biomarkers were studied in order to better understand the complex mechanisms leading to greater cardiovascular risk in originally healthy but more hostile individuals. Finally, in primary and post-hoc analyses, we controlled for pertinent behavioral, psychological, physiological, and medical variables, which allowed us to disconfirm the hypotheses whereby sex and age differences in sexual hormones or autonomic and neuroendocrine responses to stress could be responsible for the observed findings.

## Conclusions

In summary, our study contributes novel information regarding the influence of hostility on acute stress-induced changes in inflammatory activity, and in particular on Il-6 and Il-10, as well as the moderating role of age and sex on these relations. Stress induced elevations in pro-inflammatory activity in young hostile individuals, as well as decreases in anti-inflammatory activity in hostile women may put them at greater risk for CAD, in much the same way as cardiovascular or neurohumoral reactivity to stress has been shown to predict later risk of CAD [15,77–79].
